# Low‐Voltage Driven Ionic Polymer‐Metal Composite Actuators: Structures, Materials, and Applications

**DOI:** 10.1002/advs.202206135

**Published:** 2023-01-22

**Authors:** Hao Zhang, Zhaohua Lin, Yong Hu, Suqian Ma, Yunhong Liang, Lei Ren, Luquan Ren

**Affiliations:** ^1^ The Key Laboratory of Bionic Engineering Ministry of Education Jilin University Changchun 130025 China; ^2^ School of Mechanical and Aerospace Engineering Jilin University Changchun 130025 China; ^3^ Weihai Institute for Bionics‐Jilin University Jilin University Weihai 264207 China; ^4^ Department of Mechanical, Aerospace and Civil Engineering University of Manchester Manchester M13 9PL UK

**Keywords:** artificial muscles, biomimetic applications, electroactivity, ionic polymer‐metal composite, low‐voltage actuator

## Abstract

With the characteristics of low driving voltage, light weight, and flexibility, ionic polymer‐metal composites (IPMCs) have attracted much attention as excellent candidates for artificial muscle materials in the fields of biomedical devices, flexible robots, and microelectromechanical systems. Under small voltage excitation, ions inside the IPMC proton exchange membrane migrate directionally, leading to differences in the expansion rate of the cathode and the anode, which in turn deform. This behavior is caused by the synergistic action of a three‐layer structure consisting of an external electrode layer and an internal proton exchange membrane, but the electrode layer is more dominant in this process due to the migration and storage of ions. The exploration of modifications and alternatives for proton exchange membranes and recent advances in the fabrication and characterization of conductive materials, especially carbon‐based materials and conductive polymers, have contributed significantly to the development of IPMCs. This paper reviews the progress in the application of proton exchange membranes and electrode materials for IPMCs, discusses various processes currently applied to IPMCs preparation, and introduces various promising applications of cutting‐edge IPMCs with high performance to provide new ideas and approaches for the research of  new generation of low‐voltage ionic soft actuators.

## Graphical abstract

The recent development and advances in ionic polymer‐metal composite （IPMCs）are reviewed. Ion exchange membranes and conductive electrodes are first discussed, which are the components of the IPMCs sandwiched structure. Subsequently, novel preparation technology and innovative applications of IPMCs are given. Finally, current challenges in IPMCs are identified, and future perspectives are discussed.

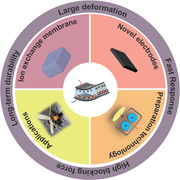

## 1 Introduction

Artificial muscles are a class of soft smart materials that respond to external stimuli and are capable of reversible deformation such as expansion, contraction, or rotation.^[^ ^1^, ^2^, ^3^ ^]^ Such deformation grants the artificial muscle the actuation function, so here, the term “artificial muscle” can be interchanged with “actuator.” Artificial muscles are expected to be used in various fields, such as biomimetic robots and biomedical devices which have proposed requirements for soft bodies.^[^ ^3^, ^4^, ^5^, ^6^, ^7^, ^8^ ^]^ To meet the development needs of soft robots, artificial muscles or actuators with different stimulus response functions have been explored, including electricity,^[^ ^9^, ^10^, ^11^ ^]^ magnetism,^[^ ^12^, ^13^, ^14^ ^]^ thermal energy,^[^ ^15^, ^16^ ^]^ fluid pressure,^[^ ^17^, ^18^, ^19^ ^]^ and light.^[^ ^20^, ^21^, ^22^, ^23^ ^]^ Among the actuators, ionic polymer‐metal composites (IPMCs), as ionic electroactive polymers, are very suitable for the development of artificial muscles because of their high energy density, large deformation, fast response, light weight, high flexibility, and amphibiousness, in addition to the advantages of precise and fast low‐voltage electrical control (generally 0.5–10 V).^[^ ^24^, ^25^, ^26^, ^27^ ^]^ Here, low‐voltage means a voltage lower than the human body safety voltage but not limited to the waveforms, which makes IPMCs highly safe for humans.

The study of IPMCs could be traced back to 1949, when certain copolymers were proposed to be able to contract and expand when stimulated by chemical means.^[^ ^28^, ^29^ ^]^ The muscle‐like properties exhibited by these polymer gels have led to the discussion of their potential as mechanochemical engines or turbines. In 1992, Shashinpoor and Oguro et al. successively proposed an actuator with electrical actuation properties obtained by plating electrodes on the surface of an ion‐exchange membrane.^[^ ^30^, ^31^ ^]^ Sadeghipour et al. also discovered the sensing properties of IPMCs in the same year.^[^ ^32^ ^]^ Since then, such electroactive artificial muscle has attracted a great deal of attention from academic researchers. In general, IPMCs have an ion exchange membrane in the middle and surface metallic electrodes. After a small excitation voltage is applied to the surface electrode, the randomly distributed hydrated cations inside the ion exchange membrane will move to the cathode side of the IPMC in a directional manner, thus making the swelling differences of the cathode and anode more obvious. The expansion on the cathode side and the contraction on the anode side cause a linear bending deformation of the cantilever beam‐shaped IPMC actuator. Extensive work has been carried out to study the actuation mechanism and improve the actuation performance, including the exploration of novel ion exchange membranes, electrode materials, and preparation technologies. The ion exchange membrane is the carrier for ion transport. Two perfluorinated polymers designed for fuel cell applications, perfluorosulfonic acid (Nafion) and perfluorocarboxylic acid (Flemion), are widely used in IPMC actuators, as they are easily manufactured and commercialized and have good performance. In addition, the conductivity of the electrode and the interface between the electrode and the ion exchange membrane largely influence ion transport and storage, which is critical to the ability to drive IPMC deformation and maintain deformation. For the electrodes, precious metals, such as gold, silver, platinum, palladium, etc., initially assumed the main task as IPMC electrodes. However, with the development of carbon materials and conducting polymers, many nonmetallic electrodes have emerged, and precious metal electrodes have been replaced by other nonmetallic electrode materials with high conductivity and flexibility. Due to the change in the combination of different materials, several names have appeared, such as ionic polymer‐conductor composite (IPCC), ionic conducting polymer gel film (ICPF), and ionic polymer transducer (IPT).^[^ ^27^ ^]^ However, IPMCs are gradually becoming a generic or surrogate term for this class of electroactive polymers, in which ion transport plays an essential part in the actuation behavior.

**Figure 1:**
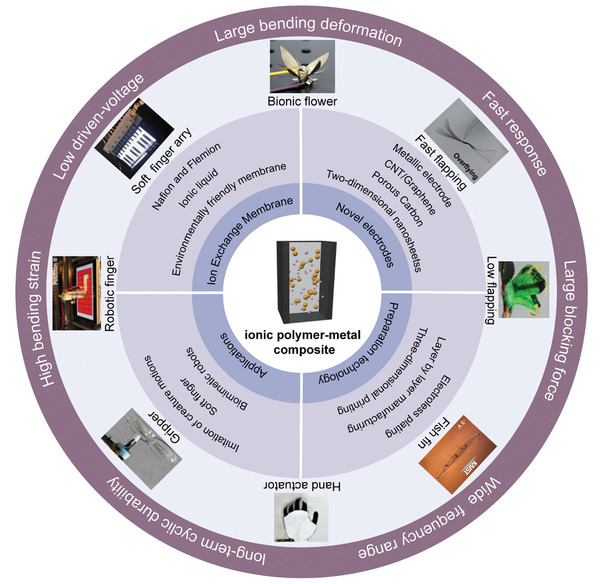
Schematic illustration of the compositions in ionic polymer‐metal composites and their applications. The bionic flower and inspired dragonfly. Reproduced with permission.^[^ ^33^ ^]^ Copyright 2019, John Wiley and Sons. The inspired butterfly. Reproduced with permission.^[^ ^34^ ^]^ Copyright 2022, American Chemical Society. The fish fin. Reproduced with permission.^[^ ^35^ ^]^ Copyright 2018, John Wiley and Sons. The hand actuator and mechanical gripper. Reproduced with permission.^[^ ^36^ ^]^ Copyright 2019, John Wiley and Sons. The robotic finger. Reproduced with permission.^[^ ^37^ ^]^ Copyright 2019, John Wiley and Sons.

The full paper is divided into four sections. As shown in **Figure** **1**, after the introduction, the first section provides a basic description of the ion exchange membranes for IPMC flexible actuators, including commonly used membranes, membrane blending and enhancement, and environmentally friendly membranes with biocompatibility. The second section focuses on the research progress in electrodes, such as conventional noble metal materials, carbon‐based materials, and their modifications. The third section reviews the various preparation methods of the IPMC actuators. Then, the applications of IPMC actuators are summarized and introduced systematically in the fourth section. Finally, future research and potential applications of IPMC actuators are discussed.

## 2 Ion Exchange Membranes for IPMC

### 2.1 Nafion and Flemion

**Figure 2:**
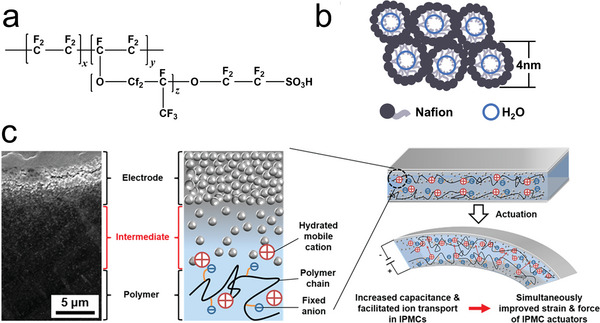
a) Chemical structure of Nafion with a hydrophobic fluorocarbon backbone and hydrophilic sulfonic acid groups. b) Schematic diagram of the parallel water channels of Nafion. c) Scanning electron micrograph and schematic diagram of the metal electrode‐polymer electrolyte interfaces in IPMC and the mechanism of the bending motion of the IPMC actuator caused by cation migration. c) Reproduced with permission.^[^ ^44^ ^]^ Copyright 2017, American Chemical Society.

Nafion, or perfluorosulfonic acid ion exchange membrane, was invented by DuPont in the 1970s and was initially used in new energy fields such as fuel cells.^[^ ^38^, ^39^ ^]^ Nafion has an interconnected network structure consisting of a hydrophobic fluorocarbon backbone and hydrophilic sulfonic acid groups (**Figure** **2**a). The hydrophilic domain surrounded by the sulfonic acid groups is capable of forming an ion channel that can reach an average size of 2.4 nm (Figure 2b), effectively absorbing solvents and providing a pathway for ion transfer.^[^ ^40^, ^41^ ^]^ Dominated by hydrated cation migration, the bending deformation process of IPMC was thought to involve the ion cluster flux and the electroosmosis of water inside Nafion. In IPMCs, anions and cations with neutralization are concentrated in ionic clusters in the hydrophilic domain. After applying a voltage, the ions are redistributed under the effect of an electric field (Figure 2c). The hydrated cations concentrate at the cathode of the IPMC, causing the clusters on the cathode side to expand and the long polymer chains to stretch. In contrast, the clusters on the anode side contract, and the unbalanced pressure caused by the difference in water content contribute to the bending motion of the IPMC. The migration and accumulation of ions allow the IPMC to be viewed as a double layer parallel plate capacitor with a coupled electrochemical‐mechanical response. The fast response and bending deformation are often followed by a slow back relaxation phenomenon and are much more pronounced under large voltages than under small voltages,^[^ ^1^, ^2^ ^]^ because the pressure in the cation‐rich region on the cathode side causes water molecules to diffuse out of this region, causing a small reverse deformation of the IPMC toward the cathode after reaching the maximum displacement. Netmat‐Nasser proposed a model of IPMC‐driven deformation that incorporates the combined effects of hydrodynamic, electrostatic, osmotic, and elastic forces.^[^ ^42^ ^]^ During the test, cations were found to continue to accumulate at the cathode even during the backward relaxation phase, indicating that the electrostatic force of the IPMC is more advantageous than the electro‐osmotic and hydraulic pressure effects. Moreover, the chemical composition and structure of the ionic polymer, the morphology of the metal electrode, the nature of the mobile cations, and the level of hydration (solvent saturation) were identified as important factors affecting the actuation behavior of IPMC.

Another common commercial ion exchange membrane is Flemion, which differs from Nafion in that the sulfonic acid group within the polymer changes to a carboxylic acid group, but is also hydrophilic. Netmat‐Nasser and Wu compared the properties of Nafion and Flemion and their corresponding IPMCs performance.^[^ ^43^ ^]^ The results showed that Flemion ionomers have higher ion exchange capacity, higher stiffness, and better water absorption than Nafion. In addition, Flemion‐based IPMCs do not undergo the backward relaxation that occurs in Nafion‐based IPMCs during the deformation process at DC voltage, but instead, undergo a slower but still cathodically oriented bending deformation.

### 2.2 Ionic liquid

In the conventional Nafion‐based IPMC, water, as a solvent, will naturally evaporate and electrolyze during the operation of the IPMC (theoretically larger than 1.23 V). In this way, the ion flux will be greatly reduced by solvent loss, and back relaxation is difficult to solve, so the stability and durability of the IPMC can hardly be maintained.^[^ ^26^, ^45^ ^]^ Therefore, some alternative solvents have been proposed for application in IPMC actuators. Nemat‐Nasser proposed ethylene glycol or glycerol as internal solvents, and the solvent uptake and retention of the IPMC and the operating time were improved.^[^ ^46^ ^]^ However, these polar organic solvents can cause a significant reduction in actuator actuation speed and limit the actuation performance. Recently, ionic liquids that have advantages such as rigid chemical structure, high stability, wide potential window, high conductivity, low viscosity, and low vapor pressure have been considered excellent alternatives to Nafion internal aqueous solvents.^[^ ^47^, ^48^, ^49^, ^50^ ^]^ Ionic liquids exist in liquid form at or above room temperature and consist of anions and cations of widely varying sizes, which are responsible for the actuation motion of the IPMC.^[^ ^40^, ^50^, ^51^, ^52^ ^]^ In addition, ionic liquids themselves are ionically conductive and would be expected to promote ionic movement within the Nafion membrane. Highly stable ionic liquids eliminate the problems of electrolysis and evaporation when water is used as a solvent. The larger potential window (normally ≈4 V) also makes larger drive voltages possible, as larger excitation voltages are generally seen to be able to drive better motion of IPMCs. Doyle et al. reported that the ionic conductivity of Nafion was greatly enhanced by the presence of ionic liquids and that the absorption of ionic liquids within the exchange membrane was proportional to temperature.^[^ ^53^ ^]^ Bennett et al. noted that the 1‐ethyl‐3‐methylimidazolium trifluoromethanesulfonate ionic liquid is a viable solvent for the IPMC transducer, demonstrating the advantages of long‐term stability in air over water‐solvated ones.^[^ ^48^ ^]^ According to Akle et al., the use of ionic liquids allows the IPMC to operate in air for more than 250 000 cycles.^[^ ^47^ ^]^ Lee et al. explored four ionic liquids as internal solvents with the same 1‐ethyl‐3‐methylimidazolium (EMIm) cation and different anions including bromide (Br), nitrate (NO_3_), acetate (AcO), and trifluoroacetate (TA).^[^ ^54^ ^]^ The experiments indicate that EMImBr and EMImNO_3_, which exhibited low electrochemical impedance, high anionic mobility, and high thermal stability exhibited larger bending deformation. The size and ion mobility of the anions are thus believed to largely influence the performance of ionic liquid‐based IPMCs. However, an existing problem is that ionic liquids, of which there is a wide variety, generally diffuse very slowly into ion exchange membranes, requiring long periods of impregnation and high temperature environments to obtain as much solvent immersion as possible. Known methods include the removal of water remaining in the IPMC due to the electroless plating process, immersion in a mixture of methanol and ionic liquid for sonication, and the necessary temperature control. Currently, many dry actuators have chosen ionic liquids as internal solvents, considering the priority of IPMC stability and durability. With the development of Nafion dispersion, a way of doping ionic liquids directly into the dry actuators has been employed, which reduced manufacturing time of the preparation for actuators.^[^ ^55^, ^56^, ^57^ ^]^

### 2.3 Environmentally Friendly Membrane

**Figure 3:**
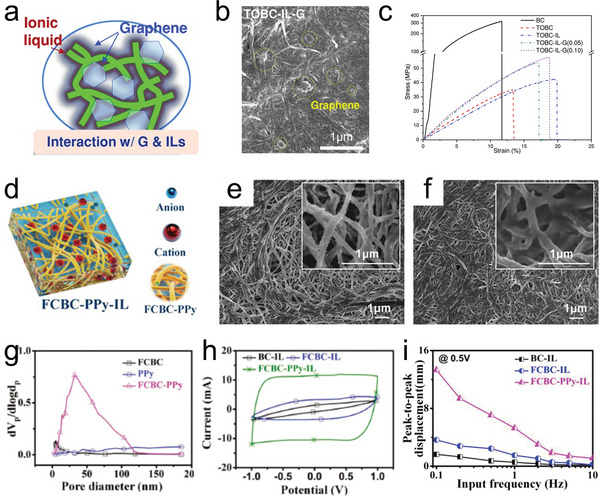
a) Schematic diagram of TOBC‐IL‐G. b) Scanning electron microscope (SEM) image of TOBC‐IL‐G. c) Tensile curves of BC and TOBC‐IL‐G composite membranes. d) Schematic diagram of the FCBC‐PPy‐IL ionically crosslinked nanocomposites. e) SEM image of FCBC‐PPy. f) SEM image of FCBC‐PPy‐IL. g) CV curves of the BC‐IL, FCBC‐IL, and FCBC‐PPy‐IL actuators based on PEDOT:PSS/DMSO electrodes under a scan rate of 100 mV s^−1^ in the potential window of +1.0 to −1.0 V. h) Specific capacitance values of the actuators under a scan rate of 100 mV s^−1^ in various potential windows. i) Peak‐to‐peak displacements of the actuators at frequencies ranging from 0.1 to 10 Hz under a sinusoidal input voltage of ±0.5 V at 0.1 Hz. a–c) Reproduced with permission.^[^ ^64^ ^]^ Copyright 2015, John Wiley and Sons. d–i) Reproduced with permission.^[^ ^66^ ^]^ Copyright 2020, John Wiley and Sons.

Although the development of Nafion is highly mature, and Nafion is used in many fields, IPMC based on Nafion still has disadvantages such as low blocking force and low actuation bandwidth, and Nafion is also a hazardous fluorinated polymer. In addition, some IPMC actuators were supposed to be used as surgical tools, ocular muscles, and heart compression devices. These application scenarios place a demand on the biocompatibility of actuator materials and some natural polymers were considered. Bacterial cellulose was proposed as a material source for ion exchange membranes for fuel cells due to its environmental compatibility and thus has the potential to be applied to IPMC.^[^ ^58^, ^59^, ^60^, ^61^, ^62^, ^63^ ^]^ Oh et al. developed bacterial cellulose actuators that were able to operate under hydrated conditions.^[^ ^60^ ^]^ However, excessive swelling of the bacterial cellulose and peeling of the metal electrode from the substrate made the actuator performance unsatisfactory. Then, Oh et al. proposed a biofriendly membrane containing 2,2,6,6,‐tetramethylpiperidine‐1‐oxyl radical‐oxidized bacterial cellulose (TOBC), chemically modified graphene, and ionic liquid for a bioelectronic muscular actuator (TOBC‐IL‐G).^[^ ^64^ ^]^ The TOBC‐IL‐G (**Figure** **3**a–c) achieved higher ionic conductivity, ion exchange capacity, and capacitance because of the provision of abundant protons from carboxylic acid groups in TOBC, together with the larger ion‐exchange sites and large surface area from the composite membrane. According to the report, the aggregation of the BC composite matrix was disrupted due to the TOBC, purification of chemically modified graphene, and plasticizing effect of IL, and a well‐dispersed biofriendly composite membrane was obtained. The TOBC‐IL‐G membrane exhibited a dramatic increase in tensile modulus (up to 23%) and tensile strength (up to 75%) compared to pure TOBC. The TOBC‐IL‐G actuator shows large deformation, fast response, and highly durable harmonic actuation. Kim et al. prepared novel electroactive hybrid actuators using freeze‐dried bacterial cellulose (FDBC), ionic liquid, and poly(3,4‐ethylenedioxythiophene)‐poly(styrene sulfonate) (PEDOT:PSS).^[^ ^65^ ^]^ On the one hand, ionic liquids were used instead of water to obtain better actuation performance in air. On the other hand, FDBC alleviates the problem of pure bacterial cellulose which has difficulty absorbing sufficient ionic liquid due to its tight structure and high crystallinity. Wang et al. designed a high‐performance low‐cost actuator based on functional carboxylated bacterial cellulose (FCBC) and polypyrrole (PPy) nanoparticles with ionic liquids as mobile ions, exhibiting large bending strain, good bending durability, wide frequency bandwidth, and high energy density.^[^ ^66^ ^]^ Carboxyl groups on FCBC enhanced the adsorption of PPy, forming a conductive network structure with pores. The biocomposite membrane of FCBC‐PPy‐IL (Figure 3d–i) exhibits a large surface area and high porosity, abundant microporosity and mesoporosity as well as strong ionic interactions between nanoparticles and functional groups, significantly improving the electrochemical properties of the actuators and thus promoting ion movement in the biocomposite membrane matrix.

As the other component of IPMCs, the intrinsic properties of electrodes with complicated structural characteristics also affect the electromechanical performance of actuators. Tiny changes in the microstructure of electrodes may influence their conductivity, capacitance, and mechanical properties, which have a complex relationship with the actuation performance. From the perspective of stratification, the increase in the passage rate of ions in the electrolyte membrane and the promotion of the storage space for charge in the electrode facilitate the fast response and large deformation of the IPMC. A large strain means that the response rate has difficulty keeping up with the change in the excitation voltages. Thus, the current challenge for high‐performance IPMCs is to achieve larger deformation and faster response simultaneously under low voltage, generally viewed as mutually exclusive properties. Hence, it is necessary to provide a systematic overview of the achievements of the electrodes for IPMC actuators and a detailed analysis of the limiting factors for achieving high actuation performance.

## 3 Novel Electrodes

### 3.1 Metallic Electrodes

**Figure 4:**
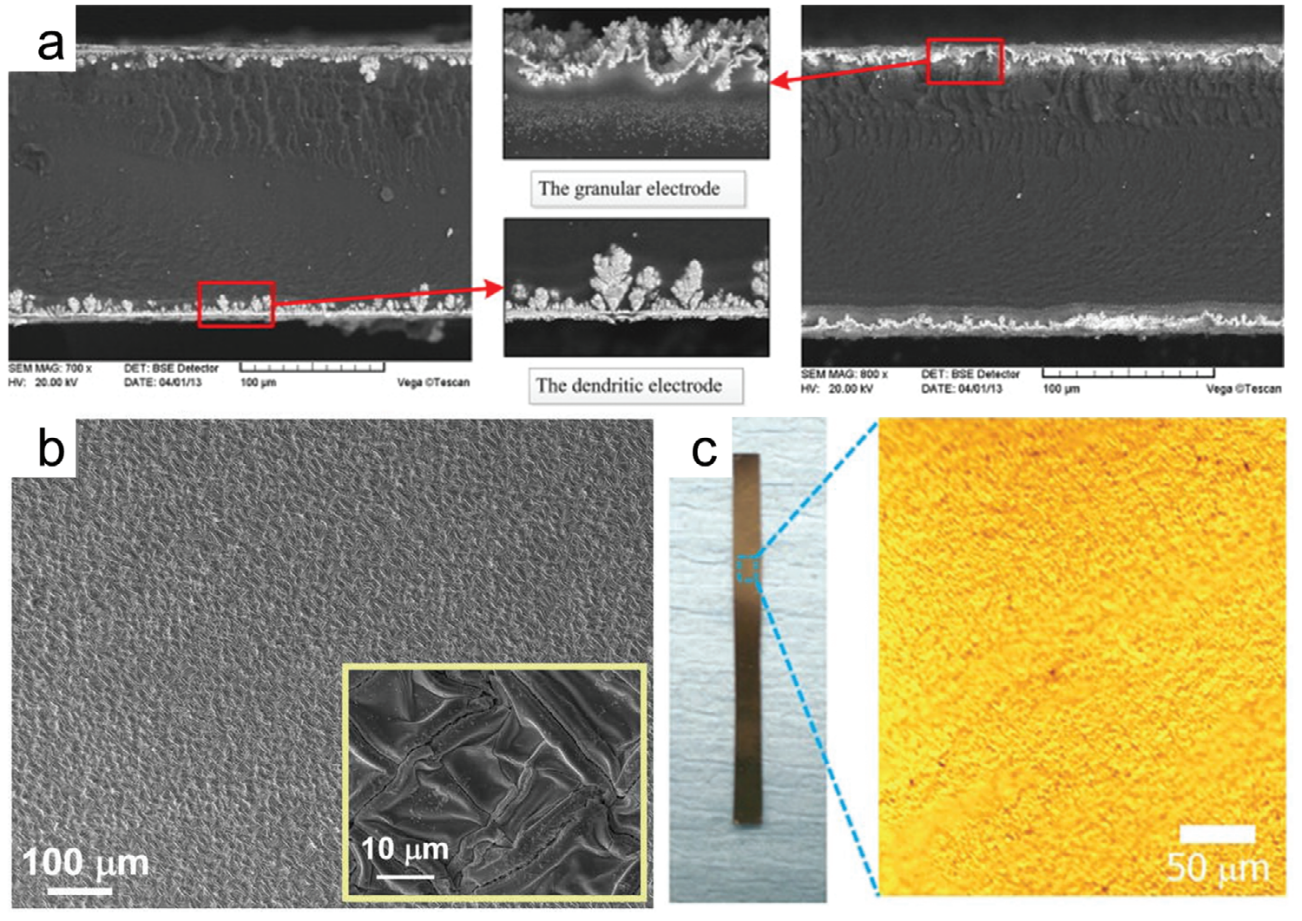
a) Cross‐section morphology of Au‐IPMC (left) and Pd‐IPMC (right). b) Surface morphology of the Pt electrode layer fabricated on the Nafion film. c) Photograph of an IGMN‐based actuator. The zoom shows an optical microscopy image of the metalized surface of the actuator. a) Reproduced with permission.^[^ ^67^ ^]^ Copyright 2014, Elsevier. b) Reproduced with permission.^[^ ^33^ ^]^ Copyright 2019, John Wiley and Sons. c) Reproduced with permission.^[^ ^71^ ^]^ Copyright 2017, John Wiley and Sons.

A major strategy has been developed in which the electrode layer of IPMC should have high flexibility and high electrical conductivity to help the whole IPMC actuator achieve high curvature deformation along the film. A high conductivity electrode is a guarantee for the formation of an electrophysical field, while high flexibility is for better performance, as high stiffness will inhibit the deformation of the IPMC. In addition, the junction interface between the electrode and the ion‐exchange membrane should ensure high adhesion but also provide as much ion‐accessible area and attachment sites as possible to obtain a better electrochemical‐mechanical response. Wang et al. compared the difference in electrode morphology between Au‐IPMC and Pd‐IPMC (**Figure** **4**a) and found that the gold electrode has a smooth surface and a dense interior and forms a dendritic structure in the ion membrane.^[^ ^67^ ^]^ The Pd electrode, however, exhibited a high diffusion of metal particles into the ion exchange membrane. Both electrodes exhibited a high specific surface area and some degree of diffusion, but Au‐IPMC had a lower surface resistance of 0.335 Ω sq^−1^ compared to Pd‐IPMC (2.64 Ω sq^−1^) while in a fully hydrated condition. Ma et al. reported IPMC with a high quality Pt electrode obtained by a novel isopropanol‐assisted electroless plating method.^[^ ^33^ ^]^ The electrode layer showed a compact nano‐ and homo‐dispersed morphology (Figure 4b). Yan et al. used the supersonic cluster light transfer (SCBI) technique to fabricate 30–100 µm thick gold thin film electrodes (Figure 4c) on the surface of polymer films, providing low surface resistance (95 Ω cm^−2^ at 100 µm) and a large surface area for more efficient charge storage. In this regard, the selection and control of the electrode thickness is a trade‐off between the IPMC electroactivity and the overall stiffness of the composite.

Pt‐based IPMC, as the earliest proposed actuator, plays a seminal role in the development of IPMCs. However, Pt electrodes were found to be susceptible to microcracks in dry environments or after multiple actuation deformations, which not only caused further water loss affecting the hydration level of the IPMC but also increased the surface resistance of the electrode and significantly decreased the conductivity. Considering this situation, the method of depositing a layer of highly conductive dielectric on the surface of a Pt electrode was proposed.^[^ ^68^ ^]^ After tuning the saline solution (Ag/Cu) concentration, deposition time, and temperature, a silver/copper coating (1–2 µm) was deposited on the Pt electrode surface of the IPMC, exhibiting a lower surface resistance and greater output force (10–20%). Currently, composite electrodes are still a viable method to enhance the performance of IPMC, and a series of composite electrodes for IPMC has been developed, including Pd‐Pt IPMC, Pt‐Ag IPMC, and the use of some 2D carbon materials, which will be described in detail in the next section. Another influence on the electrode conductivity is the size of the nanoparticles. Although not obvious on the macroscopic scale, the small size of metal electrode nanoparticles on the microscopic scale makes it easier to form a uniform, smooth electrode layer. The size of Pt particles at the boundary of the IPMC electrode is ≈40–60 nm, and the size of the condensed particles is much higher than the size of incipient particles (≈5 nm). Shahinpoor and Kim proposed a method to enhance the dispersion of Pt ions within the ion‐exchange membrane by adding appropriate dispersants during the electroless plating process to reduce the generation of large condensed particles.^[^ ^26^ ^]^ High‐quality nano‐dispersed electrodes were obtained, which on the one hand reduced the electrode surface resistance and on the other hand were able to reduce water leakage, allowing the IPMC to exhibit faster response and higher force density. For example, the addition of different alcohol solutions for assistance during the electroless plating process was proposed to allow the Nafion membrane to swell more efficiently during the reduction step.^[^ ^69^, ^70^ ^]^ The metal salt ions achieve a better distribution on the surfaces of the substrate in this way. The high‐quality, homogeneous nano‐dispersed Pt electrodes obtained became the structural basis for the large deformation and fast response of IPMC. Nevertheless, traditional precious metal materials for IPMCs still have some limitations such as time‐consuming processes, environmental unfriendliness, and high cost due to limited metal resources.

For the electrode layer, a thick metal layer will greatly increase the stiffness and elastic modulus of the IPMCs and crack easily, which will seriously affect the operation of IPMCs. Moreover, the loss of water has not been completely solved, as the water content is critical for long‐term operation of the IPMCs. These inherent problems prompted the search for electrode materials with higher flexibility and excellent electromechanical properties, which are of great significance for the next generation of high‐performance ionic soft actuators.

### 3.2 CNT/Graphene

Since the discovery of multiwalled carbon nanotubes (MWCNTs),^[^ ^72^, ^73^ ^]^ carbon nanomaterials such as carbon nanotubes (CNTs)^[^ ^74^, ^75^, ^76^, ^77^ ^]^ and graphene^[^ ^78^, ^79^, ^80^ ^]^ have received much attention in various fields for their unique mechanical and electrical properties. Interestingly, CNTs and graphene are promising candidates for electrodes of IPMC actuators, where conductivity and flexibility are needed. Multiwalled carbon nanotubes have a tubular structure composed of several layers of graphene, with outer diameters ranging from 3 to 30 nm. Their excellent properties are derived from the sp^2^ hybridization of carbon atoms in graphene, where each atom is uniformly connected to three carbon atoms in the plane (120°), exhibiting a hexagonal honeycomb lattice with a weak *π*—bond on the *z*–axis. The later discovered single walled carbon nanotubes (SWCNTs), however, are a single atomic layer of graphene rolled into a circular tube, exhibiting an extremely large aspect ratio (≈1000), and are therefore considered to be 1D materials. In contrast, graphene nanosheets are considered 2D materials, with dense nanostructures exhibiting porosity and ultrahigh ion‐accessible surface area (specific surface area). With 1D CNTs and 2D graphene proving promising in IPMC actuators and novel 3D electrodes being proposed, carbon nanomaterials are increasingly being used to fabricate ion soft actuators. In 1999, Baughman et al. reported a bimorph cantilever actuator based on SWCNTs that could operate in electrolytes and ions could be inserted into the electrode.^[^ ^74^ ^]^ Thus, the nanostructure of carbon nanomaterials is able to provide an additional space for the insertion of mobile ions inside the IPMC, exhibiting advantages not found in conventional metallic electrodes.

**Figure 5:**
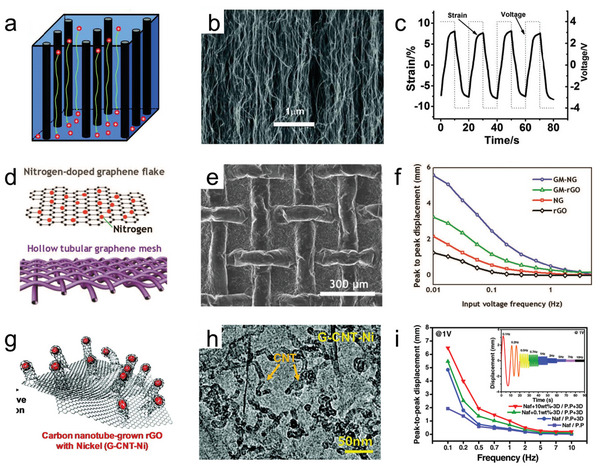
a) Schematic diagram of the direct ion transport paths in VA‐CNT/Nafion CNCs. b) SEM image of the CNT forest. c) Strain of the IPCNC actuator versus time under an AC square‐wave voltage. d). Schematic diagram of the nitrogen‐doped graphene flake and the hollow tubular graphene mesh. e) SEM image of the GM‐NG hybrid electrode surface. f) Bending displacements of GM‐NG actuators under different frequencies. g) Schematic diagram of the 3D G‐CNT‐Ni heteronanostructures. h) TEM images of the G‐CN‐Ni heteronanostrucures. i) Bending displacement of the G‐CNT‐Ni‐based actuators at different frequencies. a–c) Reproduced with permission.^[^ ^81^ ^]^ Copyright 2010, John Wiley and Sons. d–f) Reproduced with permission.^[^ ^82^ ^]^ Copyright 2017, John Wiley and Sons. g–i) Reproduced with permission.^[^ ^56^ ^]^ Copyright 2017, John Wiley and Sons.

Liu et al. used a modified chemical vapor deposition method to fabricate highly vertically aligned carbon nanotubes (VA‐CNTs) for electrodes and mechanically densified them to preserve the alignment and uniformity of CNT packing (**Figure** **5**a,b).^[^ ^81^ ^]^ The VA‐CNTs were mixed with Nafion to form a conductive network composite (CNC) and combined with IL/Nafion ionomer to form the actuator (IPCNC). As a result, the thicknesses of the CNC, ionomer, and actuator were 12, 25, and 49 µm, respectively. The anisotropic CNCs enabled the suppression of unwanted strain (thickness direction) in the actuator. As shown in Figure 5c, a high bending strain (>8% under 4 V) was obtained. According to the report, the morphological control of CNTs and thus the enhanced electroactive device performance is reflected in three aspects: the channels between VA‐CNTs provide a continuous path for fast ion conduction, the continuous CNTs reduce the electrical conduction resistance, and the creation of the desired elastic anisotropy enhances the actuation strain. As reported above, the controlled structure of 2D nanomaterials provides high‐speed channels and charge storage sites for ion migration, which facilitates the overaccumulation of electrolyte ions. However, 2D nanomaterials are susceptible to restacking by van der Waals forces and film‐forming properties, leading to a significant reduction in specific surface area, closure of ion channels, and hindered ion diffusion, which adversely affects the performance of the actuator. Therefore, advanced nanostructures have been further developed and designed, such as porous skeletons, ordered networks, and array interfaces. In addition, multicomponent material hybrids (high conductivity, pseudocapacitive activity, mechanical flexibility) have been proposed to obtain better electromechanical properties. The optimization of the structure and composition aims to provide better transfer pathways and smoothly directed active channels for ion migration and charge storage. A functionally antagonistic hybrid electrode (Figure 5d,e) was reported by Tabassian et al., consisting of hollow tubular graphene meshes (GM) and nitrogen‐doped crumpled graphene (NG).^[^ ^82^ ^]^ GM, as the outer layer of the electrode, has a high conductivity (6.38 × 10^4^ S cm^−1^) and low capacitance favoring charge transfer, while inner NG has a low conductivity and high capacitance favoring charge storage. The functions of the two are antagonistic, but the specific properties of each exhibit synergistic effects for the whole actuator. The prepared GM‐NG electrode exhibits lower surface resistance (13.26 Ω sq^−1^) and better capacitance than the reduced graphene oxide (rGO) electrode. The actuation performance of the GM‐NG based actuator was significantly improved (Figure 5f), including 620% displacement, ten times faster rise time, and lower phase delay. Hence, the synergistic effect of GM and NG was demonstrated, and the NG wrinkles were reported to not only provide an ion‐accessible surface and excellent mechanical stretchability, but also to effectively prevent aggregation and restacking of nanosheets. However, the electron transfer in 2D nanostructures is unidirectional or bidirectional,^[^ ^83^ ^]^ while the construction of a 3D interconnected electronic network would provide more pathways for ion transfer and further promote the diffusion and accumulation of ions. Kim et al. proposed 3D graphene‐carbon nanotube‐nickel (G‐CNT‐Ni) heteronanostructures (Figure 5g,h) via microwave irradiation, which exhibited excellent electrochemical properties.^[^ ^56^ ^]^ Such a conductive network with a high surface‐to‐volume ratio and porous structure can effectively prevent the restacking phenomenon of 1D carbon nanotubes and 2D graphene. High‐performance G‐CNT‐Ni heteronanostructures as nanofillers were blended with PEDOT:PSS for the preparation of electrodes. Similarly, the heteronanostructures were also homogeneously dispersed in Nafion dispersion, and the ionic liquid was simultaneously added to the mixture and cast to obtain a blended ion‐exchange membrane with enhanced ionic conductivity and mechanical stiffness. Finally, the electrode solution was cast successively on both surfaces of the ion‐exchange membrane to form a sandwich actuator supported by a 3D heterostructure in the thickness direction. The G‐CNT‐Ni heterostructure is able to connect the amorphous regions inside the semicrystalline polymer Nafion, where the oxygen is electronegative and can attract mobile ions and facilitate their transfer. The interaction between the functional groups of the heterojunction and the dissociated ions of the ionic liquid under the electric field promotes the ionic conductivity. The 10 wt% of the Nafion/G‐CNT‐Ni composite film exhibits a mechanical strength of 69.28 MPa and an ionic conductivity of 0.254 S m^−1^ without electroosmosis, that is, the composite film maintains its insulating properties. In terms of actuation performance, the Nafion/G‐CNT‐Ni (10 wt%) composite actuator with PEDOT:PSS/G‐CNT‐Ni electrodes was demonstrated, including 6.59 mm bending displacement (Figure 5i), 2.37 times the hysteresis force, and excellent durability with up to 4 h of cyclic operation without significant degradation. The high electrochemical‐mechanical properties of 3D G‐CNT‐Ni heteronanostructures result in a beneficial synergy between the actuator components.

### 3.3 Porous Carbon

**Figure 6:**
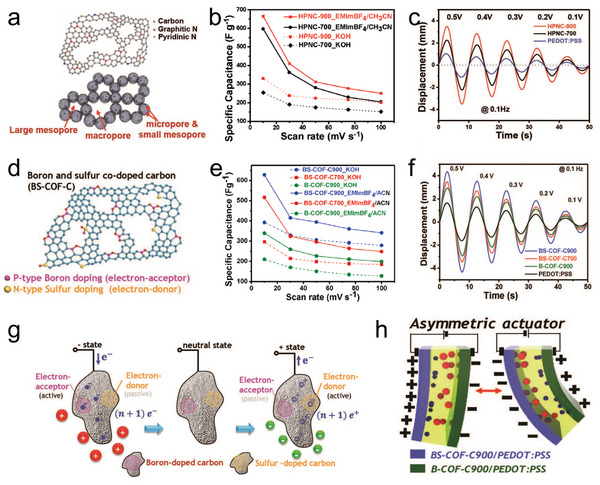
a) Schematic diagram of hierarchically porous nitrogen‐doped carbon (HPNC). b) Specific capacitance values of HPNC‐700 and HPNC‐900 at various scan rates. c) Bending performances of the electrodes under various peak voltages (0.1–0.5 V) at 0.1 Hz. d) Schematic diagram of boron and sulfur co‐doped carbon (BS‐COF‐C). e) The variation in specific capacitances with scan rates in both electrolytes. f) Bending responses of the actuators under varying sine waves. g) Effect of electron mobility and ionic interactions under electric field for BS‐COF‐C with functionally antagonistic co‐doping. h) Schematic illustrations of the asymmetric actuation mechanism of the asymmetric ionic actuator. d–h) Reproduced with permission.^[^ ^94^ ^]^ Copyright 2019, John Wiley and Sons.

With the development of research in carbon materials, porous carbon has become a cost‐effective material for energy storage due to its wide accessibility and large‐scale preparation.^[^ ^84^, ^85^, ^86^ ^]^ In addition to the excellent electrical conductivity, the feasibility of porous carbon as an electrode material is derived from its microscopic pore structure with a large specific surface area, which facilitates electrolyte penetration and ion diffusion. Moreover, heteroatom doping approaches (nitrogen, sulfur, boron, and phosphorus) have been proposed in recent studies as an effective strategy to tailor the electrochemical activity of carbon materials.^[^ ^87^, ^88^ ^]^ The large size of atoms is favorable to the generation of more charge active sites and can promote fast electron transfer, high charge capacity, and high electrolyte accessibility.^[^ ^89^, ^90^, ^91^ ^]^ As the actuation mechanism previously mentioned, the charge storage level of the electrode largely influences the volume change of the actuator. Therefore, heteroatom doped porous carbon is expected to promote the electrochemical‐mechanical response of IPMC actuators. Roy et al. fabricated hierarchically porous nitrogen‐doped carbon (HPNC) derived from poly (triazinetriptycene) organic frameworks via a one‐pot carbonization method (**Figure** **6**a).^[^ ^92^ ^]^ With the introduction of PEDOT:PSS as a favorable binder to obtain high capacitance (Figure 6b) and electrical conductivity, two HPNC/PEDOT:PSS electrodes were placed on both sides of the Nafion/EMIMBF_4_ membrane for the preparation of the actuator. The HPNC/PEDOT:PSS electrode exhibited a high specific capacitance of 330 F g^−1^ due to its large specific surface area (830.46 m^2^ g^−1^), and hierarchical micro‐ and mesoporous structures. Based on such electrodes, the peak‐to‐peak displacement of the actuator reached 6.99 mm under a low AC voltage of 0.5 V, which was 3.3 times higher than the peak‐to‐peak displacement of the pure PEDOT:PSS‐based actuator as shown in Figure 6c. In a 5‐h long‐term test, the actuator shows remarkable durability due to the high mechanical and electrochemical properties of the HPNC/PEDOT:PSS electrodes. In addition, HPNC/PEDOT:PSS shows high electromechanical energy efficiency thus generating a large blocking force of 0.998 mN, which was 1.98 times the blocking force of the PEDOT:PSS actuator. Thus, the organic frameworks appear to provide convenience for the preparation of functional porous carbon materials. Lu et al. developed electrochemical actuators using porous nitrogen‐doped carbon (NDC) derived from the zeolitic imidazolate framework.^[^ ^93^ ^]^ The corresponding actuator exhibited high capacitance (224.1 g^−1^) and large bending displacement (17.5 mm under 3 V at 0.1 Hz). Recently, boron and sulfur co‐doped porous carbon electrodes (BS‐COF‐Cs) were developed using a thiophene‐based boronate‐linked covalent organic framework as a template (Figure 6d).^[^ ^94^ ^]^ The boron doping part and the sulfur doping part were functionally antagonistic, as the former was an electron‐acceptor and the latter was an electron‐donor. While boron atoms introduce hole charge carriers decreasing the charge‐transfer resistance, sulfur doping creates a high electron density at the surface of the carbon, generating electron carrier and resulting in an increase in the conductivity.^[^ ^95^, ^96^ ^]^ PEDOT:PSS was used as a binder again to design the flexible composite electrode. As a result, the boron and sulfur co‐doped (BSC) electrode exhibited high specific capacitance (389 F g^−1^), high electrical conductivity (752 S cm^−1^), and excellent mechanical strength with a tensile modulus of 1664 MPa (Figure 6e). Benefitting from these properties, the BSC actuator has impressive actuation performance, including a large bending deformation of 8.6 mm (Figure 6f), bending strain of 0.62%, wide frequency response (up to 10 Hz), and 6 h durability with 90% retention under 0.5 V at 1 Hz. Moreover, the interesting asymmetric actuator consisting of B‐doped and BS co‐doped electrodes was constructed to evaluate the antagonistic functionality of the designed electrode. Greater accumulation of ions was always found at the BS co‐doped electrode compared to the other side, further satisfying the importance of the use of antagonistic electrodes for ionic artificial muscle (Figure 6g,h).

### 3.4 MXene and Black Phosphorus

**Figure 7:**
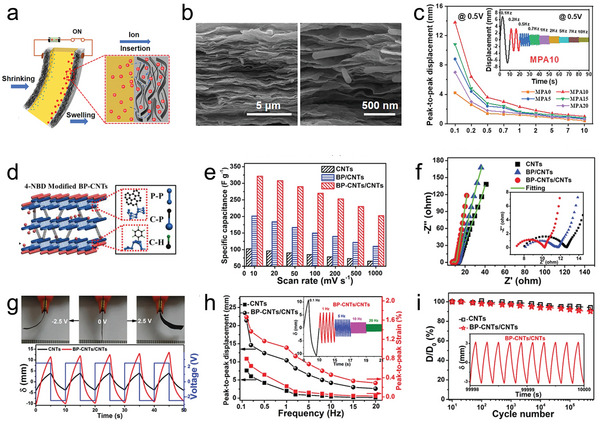
a) Schematic diagram of the ion insertion at the interface of the Nafion and MPA electrodes. b) Cross‐sectional SEM characterizations of the fracture surfaces of the ionic soft actuator MPA10. c) Bending responses at 0.1–10 Hz frequencies of the MPA‐based actuators. d) Schematic diagram of the chemically bonded BP‐CNTs electrodes. e) The calculated specific capacitances under different scan rates of different actuators. f) Nyquist plots of actuators. Inset: Nyquist plots in the high frequency region. g) Photographs of the BP‐CNTs/CNTs actuator under a voltage of ±2.5 V and the bending deformations of actuators at 0.1 Hz. h) Peak‐to‐peak displacements and strains under different frequencies. Inset: Variation in the actuation displacements with increasing frequencies. i) Cyclic stabilities of actuators under a voltage of ±2.5 V and frequency of 5 Hz. a–c) Reproduced with permission.^[^ ^34^ ^]^ Copyright 2022, American Chemical Society. d–i) Reproduced with permission.^[^ ^36^ ^]^ Copyright 2019, John Wiley and Sons.

Extensive works on actuators based on carbon‐based materials have been implemented, where ionic liquids are used to provide mobile ions. However, large ionic liquid cations have sluggish kinetics and poor transport, thus slowing the actuator response at high frequencies. Considering these limitations stemming from the insertion and extraction of ionic polymers and conductive electrode interfaces, novel electrodes are urgently needed. MXenes are a generic term for an emerging class of 2D inorganic compounds that have been widely used in the field of catalysis and energy storage.^[^ ^97^, ^98^, ^99^, ^100^ ^]^ Since their discovery in 2011, more than 30 different MXenes and their synthesis methods have been investigated.^[^ ^101^ ^]^ In general, MXenes have a general molecular formula of M*_n_*_+1_X*_n_* T*_x_*, where M represents transition metal, X represents carbon or nitrogen, and T*_x_* represents the number of surface terminal functional groups (O, OH, and F). Among these MXenes, electrically stimulated MXenes, such as Ti_3_C_2_T*_x_* and Nb_2_CT*_x_*, exhibit high electrical conductivity and high storage capacitance making them newcomers in the field of actuators. However, the stretchability of MXenes is poor, which makes the preparation of electrodes that have good adhesion with ion‐exchange membranes difficult. Therefore, various polymers have been employed as binders to maintain the conductivity and flexibility for charge storage and electrode applications.^[^ ^102^, ^103^, ^104^ ^]^ Umrao et al. developed an actuator using ionically cross‐linked Ti_3_C_2_T*_x_* as the electrode, where PEDOT:PSS chains were ionically bonded with Ti_3_C_2_T*_x_*, providing a synergistically favorable architecture for ion transport and insertion.^[^ ^105^ ^]^ The PEDOT:PSS intercalated between the Ti_3_C_2_T*_x_* layers and enhanced the porosity, which would enlarge the accessible surface area of the electrode for the ions. The results showed that the Ti_3_C_2_T*_x_*/PEDOT:PSS electrode had excellent electrical conductivity (14590.56 S cm^‐1^) and higher tensile strength (23.29 MPa) than that of human skeletal muscle (0.3 MPa). Apparently, the ionic cross‐linked electrode was beneficial for obtaining high‐performance IPMCs by enhancing the charge storage ability and ion interacting capacity. Liu et al. reported electrodes formed by a hybrid method of silver nanowires (AgNWs), Ti_3_C_2_T*_x_* MXene, and PEDOT:PSS.^[^ ^34^ ^]^ AgNWs could be interspersed in the MXene sheets to build a 3D network structure. As shown in **Figure** **7**a, the 3D network provided sufficient space for ion insertion when the actuator was deformed. Such a 3D structure was demonstrated by the SEM image of the cross‐section of the electrode (Figure 7b). The AgNWs occurred in the MXene sheets, making a larger volume for mobile ions. A high accessible surface area was beneficial to a high storage of charge capacity, and the AgNWs were believed to be able to improve the electrical conductivity. Hence, the MXene‐PEDOT:PSS/AgNWs (MPA) actuator could operate under an ultralow driving voltage. As a result, the MPA actuator exhibited large bending strain (0.48%), wide response range (Figure 7c), and great cyclability. Wang et al. prepared an MXene/Polystyrene‐Mxene hybrid layer as the electrode, because the tightly restacked structure of Ti_3_C_2_T*_x_* films makes it difficult for the ions to migrate.^[^ ^106^ ^]^ The polystyrene (PS) microspheres added into Ti_3_C_2_T*_x_* formed convenient pathways for ions to easily diffuse and aggregate in the electrode layer. The sandwiched structure of the actuator shows good interlayer adhesion between the electrode layer and the intermediate electrolyte layer. In this way, the 3D architecture formed by PS microspheres provides the actuator with unimpeded ion pathways for ionic short diffusion and fast injection. The peak‐to‐peak displacement and peak‐to‐peak strain of the MXene/PS‐MXene actuators reached 35 mm and 1.18% under a 1.5 V, 0.1 Hz square voltage, respectively. Furthermore, such actuators have a lower Young's modulus than the MXene actuators, which would be beneficial to deformation. The MXene/PS‐MXene actuators also show decent bending deformation over a wide range of frequencies and a high response rate (16.8 mm s^−1^), in which the lower Young's modulus and unimpeded ion pathways established in the electrode layer were believed to make the deformation more favorable.

Black phosphorous (BP) is also a new class of 2D nanomaterials that have been demonstrated to possess strong in‐plane bonds, weaker van der Waals interactions, and high carrier mobility.^[^ ^107^ ^]^ Wu et al. designed a high‐performance electrochemical actuator made of hierarchical structured BP‐CNTs electrodes, in which BP and CNTs are chemically bridged by P—C covalent bonds.^[^ ^36^ ^]^ As shown in Figure 7d, an in situ evaporation method was conducted under high thermal treatment to obtain hierarchically structured BP‐CNTs. The framework of BP‐CNTs provided the possibility for faster and more ion insertion/desertion, and CNTs helped to relieve interlayer restacking of BP, maintain the interlayer electron conduction (192.5 S cm^−1^) and mechanical stability (Young's modulus 952.1 MPa and break elongation 3.43%) at a favorable level. Figure 7e shows the largest capacitance (324.1 F g^−1^) of the BP‐CNTs/CNTs actuator, which is a 314% improvement over the capacitance of CNTs actuator. A pair of redox peaks is possibly related to the active interactions between phosphorus functional groups and electrolyte cations. From the Nyquist plots in Figure 7f, the BP‐CNTs electrodes have a lower contact impedance, indicating faster electron conduction in its stable surface film. Figure 9g, h presents the large bending deformation of the BP‐CNTs actuator, which reached 21.4 mm under an AC voltage of 2.5 V at 0.1 Hz. The ability of the BP‐CNTs actuator to respond in a wide frequency range was demonstrated, even at a high frequency of 20 Hz, the bending strain still remained 0.29%. Additionally, the BP‐CNTs actuator exhibited excellent durability after continuous operation for 500 000 cycles.

The performance of MXene and black phosphorus as electrode materials is enhanced by the rich pathways formed by the layered structure. Good electron conduction and redox activity facilitate the movement and diffusion of ions. The compositions of superior conductive nanomaterials and unique framework structures, which can form a skeleton with good interaction and homogeneity, are highly desirable for the enhancement of IPMC actuator's performance.

The variety of nonmetallic electrode materials applied to IPMC actuators becomes increasingly diverse as research progresses. In a comprehensive view, the overall development continues to focus on the following points: 1) Microstructural framework: Porous skeletons, large specific surface area, ion channels, and ordered structures that can provide space for ions to migrate and store, facilitating their diffusion and actuator volume changes. 2) Conductivity and electrochemical activity: The electrochemical‐mechanical response mechanism of IPMC actuators raises the need for electrode conductivity and high storage capacity, and heteroatom doping and the addition of structural materials have been demonstrated to promote charge transfer and enhance electrochemical activity. 3) Mechanical reliability: Stability is an important reference indicator for actuators, and flexibility and mechanical strength are guaranteed for IPMC to work for a long time and benefit its back‐end applications. However, when (2) and (3) are mutually exclusive, composite materials are considered an effective approach to retain the respective performance advantages, for example, PEDOT:PSS has been used as a binder for electrodes in many studies.

## 4 Selection of New Preparation Technology

The preparation of sandwiched actuators can be summarized as the following parts, namely, the preparation of ion exchange membranes, the preparation of electrode materials, the combination of ion exchange membranes and electrode materials, and the substitution of internal solvents and ions. Although the three‐layer structure of IPMC seems to be simple, there are still many factors affecting the IPMC actuation performance, such as layer thickness, interlayer bonding, strength, etc. Therefore, the methods around the actuation performance enhancement of IPMC actuators are mainly the thickness enhancement of the proton exchange membrane, the preparation of high‐quality noble metal electrodes, the deposition of high conductivity nonmetallic electrodes, the design of IPMC shape and diverse deformation, etc. One of the most critical factors is the method of electrode film formation on the ionic membrane. The method of preparing metal electrodes by electroless plating is the most commonly used and was proposed at the beginning. Based on the reduction method of electroless plating, the method of adding alcohol solution to enhance the swelling ratio for the formation of nano‐dispersed metal electrodes was proposed.^[^ ^44^ ^]^ However, the chemical method was time‐consuming, complicated, and costly, and the flexible nonmetallic electrodes developed because of the limitations of metal electrodes were widely noticed, and the method of preparing electrodes by physical casting was applied. In addition, 3D printing has gradually been proposed to prepare IPMC substrates of various shapes, which is a novel way of combining physical and chemical methods.

### 4.1 Electroless Plating

The application of electroless plating was proposed as early as 1998 by Shahinpoor et al. for the preparation of composites obtained by depositing metal layers on the surface of a polymer.^[^ ^108^ ^]^ Based on the water‐absorbing nature of the ionomer, the metal salt solution is immersed into the matrix of the ionomer. Electroless plating refers to the use of a suitable reducing agent to reduce the metal ions adsorbed on the surface of the ionomer to metal monomers and to form a metal layer covering the surface. The entire process takes place in a water bath environment at a specific temperature, so it is also known as impregnation reduction plating.

With the technical optimization of researchers, the current electroless plating technique applied to IPMC preparation can be divided into four steps: first, the pretreatment of the Nafion film (or other proton exchange membrane), including the polishing of the substrate, impurity removal, cleaning, etc. The main purpose of polishing is to increase the roughness of the Nafion film surface. Maximizing the interfacial area of Nafion makes it easy to absorb metal ions and form electrodes with high surface area. Some researchers have also proposed sandblasting and other ways to increase the roughness, but sandpaper sanding is still an efficient and simple method.^[^ ^109^, ^110^ ^]^ The impurity removal process focuses on the organic and metallic impurities on the surface of the substrate film. With the completion of cleaning, the pretreated film shows a high degree of transparency and is relatively soft. The second step is the ion adsorption process. DuPont's Nafion membranes are still the most commonly used ion exchange membranes for ionic soft actuators. The hydrophilic nature of the internal ion clusters allows them to rapidly absorb water and swell in aqueous solutions. The solution in which Nafion is immersed during ion adsorption is mostly a salt solution corresponding to the metal electrode material. As time increases, metal ions are continuously introduced into the Nafion film. The third step is the ion reduction process, which is also the most important part of electroless plating. Impregnation reduction is found to be a better method of electroless plating. Nafion is placed in water, and the reducing agent solution (e.g., sodium borohydride, potassium borohydride, etc.) is added to the aqueous environment of Nafion by adding small amounts in several drops. In this way, it is possible to avoid the reduction process becoming too violent and generating many bubbles, which can lead to uneven and discontinuous deposited electrodes. It is a longer process, but an excellent way to obtain high‐quality nano‐dispersed electrodes.^[^ ^33^ ^]^ Moreover, the number of ion adsorption and ion reduction processes has a large effect on the surface resistance and conductivity of IPMC electrodes, and we will define this number as the number of electrode layers. The number of electrode layers also affects the stiffness of this IPMC to some extent, and the high stiffness and bending actuation performance of the IPMC are mutually constrained. Therefore, two ion adsorption and ion reduction processes are generally accepted at present.^[^ ^33^, ^44^, ^69^, ^111^ ^]^ The final step is the replacement of ions that can form a larger volume of hydrated cations (such as Li ions) inside the IPMC film.^[^ ^70^ ^]^ Since the deformation of IPMC depends on the accumulation of water molecules on the mesoscopic scale, the more water molecules there are under the action of an electric field, the more pronounced the bending of IPMC will be, and the better the actuation performance will be. After up to 24 h of saturated solution immersion, ion exchange can be completed.

### 4.2 Layer‐by‐Layer Manufacturing

**Figure 8:**
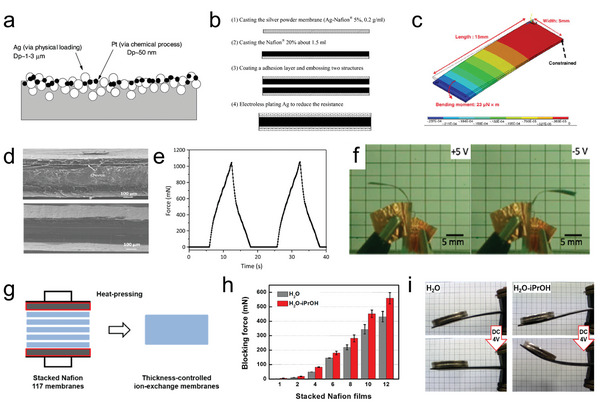
a) Schematic process illustration of the actuator with a physically loaded particulate electrode. b) The processing flow of the IPMC actuator with Ag nano‐powders. c) Simulation of the actuating behavior of the IPMC actuator with the Ag (nano‐powders)–Nafion 5% electrode. d) Cross‐sectional SEM image of the SWCNT BP/EMI^+^SCN^−^‐Nafion/SWCNT BP actuator prepared at 80 °C/60 psi (top) and 120 °C/120 psi (bottom). e) Blocking force of the composite actuator at applied voltage of ±3 V with a frequency of 50 mHz. f) Bending motion of the actuator at ±5 V with a frequency of 0.15 Hz. g) Schematic diagram for preparing ultrathick IPMC actuators based on thickness‐controlled ion‐exchange membranes. h) Blocking force of the IPMCs for different thicknesses. The IPMCs were plated in water and in the H_2_O‐iPrOH solution. The blocking force was recorded with a load cell at a point 30 mm away from the grip under a potential of 4 V of DC for 30 s. i) Actuation test of the H_2_O‐ and H_2_O‐iPrOH IPMCs with dimensions of 20 × 60 × 1.0 mm^3^ under a potential of 4 V DC for 20 s. The weight of the coins was ≈15.4 g. a) Reproduced with permission.^[^ ^112^ ^]^ Copyright 2002, Elsevier. b,c) Reproduced with permission.^[^ ^113^ ^]^ Copyright 2006, Elsevier. d–f) Reproduced with permission.^[^ ^57^ ^]^ Copyright 2017, American Chemical Society. g–i) Reproduced with permission.^[^ ^44^ ^]^ Copyright 2017, American Chemical Society.

Structurally, the preparation of IPMC consists mainly of the preparation of the ion exchange membrane substrate and the preparation of the electrodes. Electrode preparation is mostly regarded as the integration of the electrode with the substrate membrane, such as constructing the electrode coating directly on the substrate membrane. In other cases, the prepared separate electrode layers need additional methods to be synthesized with the substrate membrane, which are considered physical loading methods. Shahinpoor et al. pointed out novel IPMCs equipped with physically loaded particulate electrodes.^[^ ^112^ ^]^ As shown in **Figure** **8**a, the physically loaded Ag powder and the chemically plated Pt layer can be secured within the polymer network, thus reducing the potential intrinsic contact resistance caused by the large particles. Solution casting, or drop‐casting, is well known as an easy, convenient technique that has been widely used to prepare films. Chung et al. reported the fabrication IPMC actuator with silver nano‐powder electrodes.^[^ ^113^ ^]^ The electrode layer was fabricated using a Nafion solution with silver nano‐powders (Ag‐Naifion 5%) by casting (Figure 8b). Then, the ionic membrane was cast on the electrode. The two same structures mentioned were embossed by coating an adhesion layer, exhibiting excellent adhesion. Finally, the electroless plating Ag was applied to reduce the surface resistance. Figure 8c shows the result of the simulation of the IPMC actuator with the Ag‐Naifion 5% electrode by finite element, indicating a low elasticity modulus and large deformation. Similarly, nonmetallic electrodes and ion exchange membranes with adjustable thicknesses could be obtained via this approach. The ionic membrane materials or the electrode materials will be made into dispersions first and then cast into the mold. Finally, the dispersion will be dried at a preset atmospheric pressure and temperature. Moreover, to load the electrode layer onto the ionic exchange membrane, the electrode material dispersion could be dripped onto the prepared substrate and then dried to form a thin coating. Due to the evaporation of solvent molecules and the effect of gravity, the electrode layer is deposited on the substrate.^[^ ^55^, ^56^ ^]^ When this bonding force, which mainly relies on the gravity and material properties, is not sufficient, the hot‐pressing method is used to increase the adhesion between the substrate and the electrode layer. The hot‐presssing method requires a specific temperature and pressure. Chen et al. fabricated IPMC actuators using the hot‐pressing method to cover a carbon nanotube electrode on an ionic liquid‐Nafion membrane.^[^ ^57^ ^]^ From Figure 8d, proper pressure and temperature during hot‐pressing allow the electrodes to effectively bond with the matrix, exhibiting strong interface adhesion without crevices. This contribution is important to the actuation performance of the actuator, including a blocking force over 1 N and decent displacement (Figure 8e,f). Additionally, since the ionic materials cannot be processed by melting,^[^ ^114^ ^]^ the hot‐pressing method can also be used to thicken the ionic exchange membranes, which could greatly enhance the generating force of IPMCs. Wang et al. proposed an ultrathick IPMC with nanodispersed metal electrodes by stacking pre‐extruded Nafion films, as shown in Figure 8g.^[^ ^44^ ^]^ Metal electrodes with a nanodispersed structure are formed on the ultrathick membrane via alcohol‐assisted electroless plating, which allows increased capacitance and facilitated ion transport. Figure 8h shows the blocking force of the actuators with different thicknesses (number of stacked Nafion films). The 2300‐µm‐thick IPMC exhibited a large blocking force of 559 mN, which is 285 times the blocking force of a conventional IPMC based on the Nafion 117 membrane. The excellent output force is demonstrated in Figure 8i, where 16 coins (124 g) were lifted to a height of ≈25 mm.

### 4.3 3D Printing

**Figure 9:**
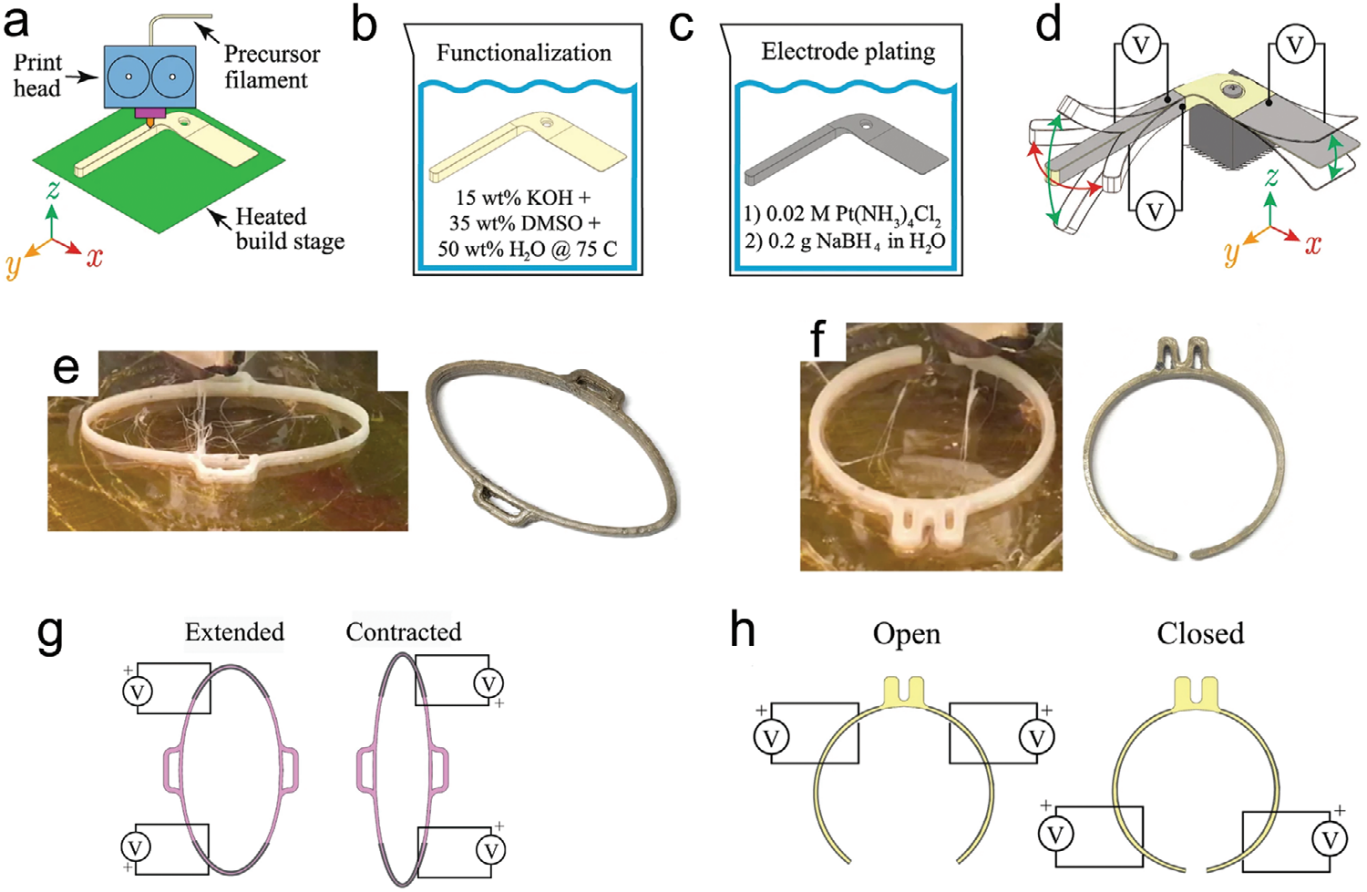
a) The printing of soft structures using precursors of ionomeric materials. b) Functionalization of the printed precursor structure. c) Electroless plating process. d) Multi‐DOF actuation of the customized actuator. e) 3D‐printed linear actuator component for modular robotic device. f) 3D‐printed gripper for the modular robotic device. g) The extension and contraction of the linear actuator. h) Open and closed states of the gripper.

Laborious electroless plating, time‐consuming laminate fabrication, and the need for costly molds make traditional manufacturing techniques somewhat limited. Currently, advances in 3D printing technology have facilitated the growth of soft materials, leading to improvements in the design and fabrication of many soft robotic systems based on smart materials.^[^ ^18^, ^115^, ^116^, ^117^, ^118^, ^119^, ^120^, ^121^, ^122^ ^]^ However, smart materials are generally capable of reversible or irreversible single‐mode deformation to only one stimulus, while robotic systems often require coordination of multi‐degree‐of‐freedom (MDOF). To enable rapid development of highly customized IPMC actuators with single and MDOF, Carrico et al. proposed a 3D fused filament additive manufacturing technique (3D printing), where structures and components could be created in a layer‐by‐layer pattern for wider applications.^[^ ^123^, ^124^ ^]^ The 3D printer systems include a Nafion filament extruder in which precursor material is extruded into a thermoplastic filament for 3D printing and a 3D printer that was specially designed for Nafion precursor filaments. As mentioned before, due to the strong ionic interactions within Nafion, it cannot be melted directly for further processing. Hence, the precursor material of Nafion resin is extruded into thermoplastic filaments, fabricated into custom‐shape structures by a 3D printer, as shown in **Figure** **9**a. Then, the material was functionalized in an aqueous solution of potassium hydroxide and dimethyl sulfoxide, and finally plated on surfaces with platinum electrodes (Figure 9b–d). In this way, Carrico et al. displayed a variety of actuators designed for the fused filament fabrication process, including a bending actuator, a linear actuator, a gripper, a rotary actuator, and a MDOF actuator. Different types of actuators are able to generate different deformation patterns, reflecting the advantages of 3D printing technology. Moreover, inspired by a caterpillar, modular IPMC components were fabricated, as shown in Figure 9e,f. The former can be used as the body to extend or contract, and the latter is supposed to be the foot to open or close. These two modular components can be assembled into a crawling robot that was able to crawl over a cylindrical pipe, which will be introduced in the next section. Therefore, the 3D‐printing approach is effective in making a monolithic body for IPMC actuators, as the new designs are increasingly complex. Additionally, segmenting of the electrode surface was also considered as a method to obtain an IPMC actuator with various motions.^[^ ^21^, ^125^, ^126^, ^127^, ^128^, ^129^ ^]^ The localized excess electrode was cut off and the partitioned electrode regions of IPMC can be supplied with electrical power independently, which would make the combination of 3D printed IPMC with complex shapes and segmented electrodes more interesting. In general, the use and initial success of 3D printing technology in the field of IPMC actuators has injected more energy into their development, facilitating the realization of more complex motions with multiple degrees of freedom, multimodality, and high integration of IPMC actuators. Chemical preparation methods are time‐consuming but precise, while physical methods are easy but relatively crude, and to obtain the best combinative performance, researchers should choose according to the characteristics of the chosen materials.

In addition to the techniques mentioned above, there are also some methods used for the exploration of IPMC applications. For example, mask technology can be used to fabricate monolithic IPMC patterned electrodes with complex motion deformation. Additionally, an external package can reduce the moisture loss of the water‐based IPMC actuator, aiming to ensure their desirable practicality.^[^ ^126^, ^130^ ^]^

## 5 Applications

IPMC actuators, as soft artificial muscles with the advantages of high bending deformation, fast response, low driven‐voltage, and safety, have excellent prospects in the fields of bionic robots, biomedical devices, and wearable electronic devices. Currently, some specific imitations of animal/plant behaviors have been implemented because the deformation and bending properties of IPMC are very similar to the deformation and bending properties of living creatures in nature. These properties include wing flapping, bionic fin wiggling, bionic flower opening, and closing, etc., all of which benefit from the wide frequency response range of IPMCs. In addition, soft robotic grippers and touch fingers are fully demonstrated, with functions such as grasping, transferring, and releasing heavy objects, generating various gestures, and even playing the piano. The fine and presettable electrical control makes these examples very vivid and interesting. Recently, IPMCs have been moving toward integration and MDOF mobility. The 3D printing, masking, and laser processing technologies have enabled the development of IPMCs with custom‐shaped and patterned electrodes, and the integration of assembly and control to form flexible actuation systems is of great importance for the development of soft robotics.

### 5.1 Imitation of Plant/Animal Motions

**Figure 10:**
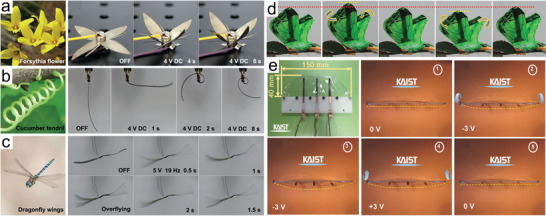
a) Images of the forsythia flower and bionic flower. b) Image of a cucumber tendril and the bionic tendril. c) Image of a flying dragonfly and bionic wing flapping based on Nafion IPMC under high frequency. d) Flapping wings of the bionic butterfly at different positions. e) Artificial fish fin based on actuators and its harmonic movement. a–c) Reproduced with permission.^[^ ^33^ ^]^ Copyright 2019, John Wiley and Sons. d) Reproduced with permission.^[^ ^34^ ^]^ Copyright 2022, American Chemical Society. e) Reproduced with permission.^[^ ^35^ ^]^ Copyright 2018, John Wiley and Sons.

The IPMC actuator with nano‐dispersed Pt electrodes prepared by Ma et al. was cut into a bionic flower with four petals and held in place by magnets connected to the positive and negative poles of the power supply.^[^ ^33^ ^]^ As shown in **Figure** **10**a, the bionic flower completes the inward bending of the petals within 8 s after a DC excitation voltage of 4 V is applied. A bionic tendril mimicking the cucumber tendril was proposed (Figure 10b). Since the IPMC was customized to a slender strip, the curling motion was achieved. Umrao et al. prepared narcissus‐like flowers using an air‐working ionic soft (AWIS) actuator based on MXene electrode.^[^ ^105^ ^]^ The six petals gradually bloomed under a square wave voltage of 3 V and the opening process of a real daffodil was very well simulated. For the imitation of animal behavior, a prototype of a flying robot inspired by dragonfly wings is shown in Figure 10c. The wing‐like vibrating motion achieves both fast directional transition and large deformation of the tip visible to the naked eye, demonstrating excellent actuation performance. Similarly, as shown in Figure 10d, Liu et al. used IPMC actuators based on silver nanowires/Mxene/PEDOT:PSS electrode to design a butterfly robot, exhibiting moving wings.^[^ ^34^ ^]^ In addition, Kotal et al. prepared an artificial fish fin that was developed with three g‐CN/NG‐based IPMCs connected in parallel and encapsulated in low‐density polyethylene (Figure 10e).^[^ ^35^ ^]^ The prototype is expected to be applied to a bionic fish robot in which variable amplitude and frequency are needed.

### 5.2 Gripper and Soft Touch Finger

**Figure 11:**
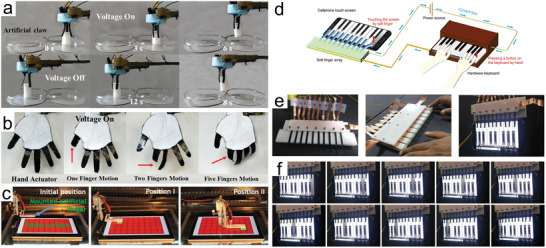
a) The artificial claw to grasp objects. b) Hand actuator to mimic finger motions. c) Soft robotic finger attached to a moving stage performing gentle touching task at designated positions on the fragile surface of a tablet. d) Schematic illustration of the working principle of the soft touch finger array for playing electronic piano applications. e) The hardware keyboard designed to control the finger array. f) Operation of all soft touch fingers for the “happy birthday” song. a,b) Reproduced with permission.^[^ ^36^ ^]^ Copyright 2019, John Wiley and Sons. c) Reproduced with permission.^[^ ^37^ ^]^ Copyright 2019, John Wiley and Sons.

A range of robotic grippers and devices that mimic human behavior have also been developed, including gestures and human‐computer interaction. As shown in **Figure** **11**a, Wu et al. designed an artificial claw with three black‐phosphorous‐based soft electrochemical actuators integrated as flexible legs. The claw grasped, transported, and released a lightweight object (153 mg), which was heavier than the gripper itself.^[^ ^36^ ^]^ The actuator based on the same material was also fabricated as a hand‐shaped robot with five mutually independent actuators, each controlled by a separate circuit (Figure 11b). Interestingly, IPMCs can also perform flexible tasks as fingers. Carrico et al. directly printed a small five‐finger hand by 3D printing.^[^ ^123^ ^]^ The electrodes on the fingers were separated, and the palm and five fingers were controlled by a total of six inputs. Manzoor et al. fabricated nanohybrid electrode (MoS2‐SNrGO) based actuators that can be used as active soft fingers to perform the required tasks directly and efficiently on the fragile surfaces of smart devices such as tablet.^[^ ^37^ ^]^ As shown in Figure 11c, the flat surface undergoes a color change at the sites touched by the actuator, while the rest of the locations still show red color. Similarly, a TP6PP ionic actuator was assembled as a piano with an array of ten robotic flexible fingers to play the piano,^[^ ^55^ ^]^ including a control part of a hardware keyboard and a corresponding part where the actuator itself is located (Figure 11d–f). The hardware keyboard is able to stimulate the corresponding actuator to undergo deformation so that it touches the corresponding piano keys on the screen of the smartphone. The above promising results demonstrate the potential of IPMC actuators and systems in the field of soft robotics.

### 5.3 Biomimetic Robots

**Figure 12:**
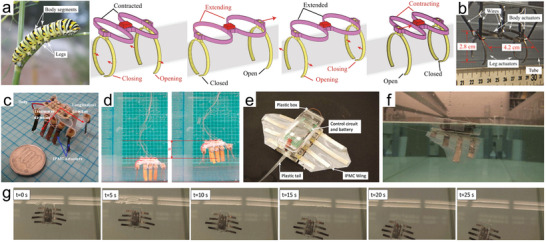
a) A 3D‐printed IPMC soft crawling robot inspired by a caterpillar and the schematic diagram of the crawling principle. b) Prototype of the 3D‐printed IPMC soft crawling robot. c) Prototype of the ten‐legged microrobot. d) The floating motion of the underwater microrobot in the water. e) A free‐swimming robotic manta with control circuits and battery. f) A snapshot of the free‐swimming robot (side view). g) The movement of the free‐swimming robot in water. c,d) Reproduced with permission.^[^ ^132^ ^]^ Copyright 2012, Elsevier. e–g) Reproduced with permission.^[^ ^133^ ^]^ Copyright 2011, Elsevier.

In addition to hand‐shaped robots, various types of bionic robots and systems that use IPMCs as actuation units have been explored. For example, Carrico et al. prepared custom‐shaped IPMCs using 3D printing and assembled them to form a caterpillar‐like crawling robot (**Figure** **12**a,b).^[^ ^120^ ^]^ The robot consists of two main parts, namely the body actuators and the leg actuators. As shown in Figure 12a, the alternating movements of the body actuator extension and contraction, with the opening and closing of the leg actuators, allow the caterpillar to walk on a tubular path with a speed of 22 mm s^−1^. Considering the nature of the IPMC that can still actuate underwater, Yeom et al. developed a jellyfish‐like robot, including a floating control part, an electrode driving part, and actual operation actuators.^[^ ^131^ ^]^ By control of the bionic input signal, the robot imitates the real two‐phase locomotion of pulse‐recovery processes of the jellyfish. The bionic input signal was found to generate a greater vertical floating displacement in the biomimetic jellyfish robot compared to sinusoidal excitation. Moreover, a biomimetic underwater microrobot with multifunctional locomotion was designed.^[^ ^132^ ^]^ The inchworm‐inspired prototype using ten IPMC actuators as legs or fingers could implement walking, rotating, floating, and grasping motions. Figure 12d shows the floating motion which could reach 6.8 mm s^−1^. Chen et al. fabricated a free‐swimming robotic batoid ray, as shown in Figure 12e,f.^[^ ^133^ ^]^ The robot consists of two artificial pectoral fins based on IPMCs, a body box containing the control circuit and battery, and a plastic tail. Each fin was assembled from a soft polydimethylsiloxane (PDMS) membrane and four separated IPMC beams to generate 3D kinematic motions including oscillation and undulation. The characterization of the robot showed a maximum twist angle of 15°, flapping deflection of 25%, tip force of 0.5 g, and low power consumption below 0.5 W. The free‐swimming process of this robot in 25 s is also shown in Figure 12g.

### 5.4 Medical Devices

In addition to the engineering applications, IPMCs have also been proposed for medical devices or biomedical applications because of their unique stimulus‐response actuation performance.^[^ ^134^, ^135^, ^136^, ^137^, ^138^ ^]^ Guo et al. proposed the application of an ionic actuator as a guide for microcatheter with two degrees of freedom in the front section as servo actuators, and the results showed that IPMC was applicable to intracavity operations.^[^ ^139^ ^]^ Lu et al. prepared a MDOF IPMC active catheter by an electrode splitting technique, aiming to obtain the deformation capability in 3D space.^[^ ^140^ ^]^ Sideris et al. designed an IPMC‐operated linear peristaltic pump, which could achieve a pumping rate of 669 pL s^−1^ (open pump).^[^ ^137^ ^]^ Moreover, a capsule‐like robot that can be used for endoscope diagnosis was proposed, using IPMCs as actuators.^[^ ^138^ ^]^ Interventional catheters, peristaltic pumps, and capsule robots are key research areas for IPMC medical applications. However, most of the descriptions of these applications are laboratory‐scale prototypes and currently exist as proofs of concept.

## 6 Conclusion

In this review, we summarize recent advances in four areas of IPMC: ion exchange membranes, electrode materials, preparation processes, and potential uses. Currently, DuPont's Nafion membrane is still the most widely used in the field of ion‐flexible actuators, including commercial membranes, dispersants, and precursor materials. However, some biocompatible ionic membranes have also been proposed, such as bacterial cellulose, given the cost and environmental considerations. Electrodes, as an important component of IPMC actuators, have a large range of choices and room for tuning. Precious metals with good electrical conductivity and ductility as traditional IPMC electrodes are being replaced by conductive polymers or 2D nanomaterials with better performance. In addition to conductivity, these materials can be designed with controllable microstructures for fast electron conduction and ion diffusion, such as heterogeneous structures and ordered networks. Likewise, the capacitive operating principle allows materials with high electrochemical activity to improve IPMC actuation performance, such as heteroatom doping carbon. The 2D nanoconductive materials provide an important way to build IPMCs with excellent electrochemical performance. In addition, during the development of the IPMCs, its preparation processes are gradually becoming refined, such as the use of isopropanol‐assisted electroless plating and the construction of 3D printing systems. More advanced preparation processes can provide assistance in the design and preparation of electrodes, or microstructures at the interface between electrodes and ionic membranes.

However, challenges remain to facilitate better performance of IPMC and drive its practical applications. First, constraints between the deformation speed, deformation size, and actuation force of IPMC still exist, and the trade‐off between these performance metrics to obtain high‐performance IPMC was, is, and will be the focus of research, along with its mechanical properties, stability, and durability. Second, the ability of IPMC actuators to achieve multiple modes of motion is the key to expanding their applicability. Finally, although some studies have been conducted on the actuation mechanism of IPMCs, microscopic studies are still lacking. Advanced in situ characterization and observation techniques will enable us to unveil the tiny actuation to further understand the relationship between internal ion diffusion and embedding, microstructural changes at the electrode interface, and macroscopic deformation. Finally, the customization of IPMC applications still must be based on solving practical problems in life. The development of many prototype devices, such as bionic flowers, artificial claws, and robots, has enabled IPMC to show great potential. Future applications tend to provide more convenience for humans in the fields of wearable devices, multifunctional robots, and human‐computer interaction systems.

## Conflict of Interest

The authors declare no conflict of interest.
